# Identification of potential novel differentially-expressed genes and their role in invasion and migration in renal cell carcinoma

**DOI:** 10.18632/aging.103192

**Published:** 2020-05-18

**Authors:** Shen-Nan Shi, Xia Qin, Shuo Wang, Wen-Fu Wang, Yao-Feng Zhu, Yu Lin, Zun-Lin Zhou, Ben-Kang Shi, Xi-Gao Liu

**Affiliations:** 1 Department of Urology, Qilu Hospital of Shandong University, Jinan, Shandong, China; 2 Department of General Surgery, Qilu Hospital of Shandong University, Jinan, Shandong, China; 3 The Graduate School of Second Military Medical University, Shanghai, China; 4 The Graduate School of Fujian Medical University, Fuzhou, Fujian, China

**Keywords:** renal cell carcinoma, *TIMP1*, biomarker, migration, invasion

## Abstract

Clear cell renal cell carcinoma (ccRCC) remains one of the most common cancer types globally, and while it has been extensively studied, the molecular basis for its pathology remains incompletely understood. Herein, we profiled three previously published datasets (GSE66272, GSE100666, and GSE105261) in a single integrated analysis aimed at identifying disease-associated patterns of gene expression that may offer mechanistic insight into the drivers of this disease. We pooled expression data from 39 normal kidney samples and 39 kidney tumors, leading us to identify 310 differentially expressed genes (DEGs) that were linked to kidney cancer in all three analyzed datasets. Of these genes, 133 and 177 were up- and down-regulated, respectively, in cancer samples. We then incorporated these DEGs into a protein-protein interaction network with the STRING and Cytoscape tools, and we were able to identify signaling pathways significantly enriched for these DEGs. The relationship between DEG expression and ccRCC patient survival was further evaluated using a Kaplan-Meier approach, leading us to identify *TIMP1* as an independent prognostic factor in ccRCC patients. When *TIMP1* expression was disrupted in ccRCC cell lines, this impaired their migratory and invasive capabilities. In summary, we employed an integrative bioinformatics approach to identify ccRCC-related DEGs and associated signaling pathways. Together these findings offer novel insight into the mechanistic basis for ccRCC, potentially helping to identify novel therapeutic targets for the treatment of this deadly disease.

## INTRODUCTION

Kidney cancer is a particularly common form of cancer, with approximately 73,820 new cases and 14,770 deaths in 2019 in the USA alone [1]. Kidney cancer is also becoming an increasingly serious problem in China, with 66,800 cases and 23,400 deaths in China [2]. The most common kidney cancer subtype is clear cell renal cell carcinoma (ccRCC), which is a particularly deadly form of urinary tumor that accounts for approximately 90,000 deaths globally each year [3]. While ccRCC has been the focus of extensive study, the exact mechanistic basis for the onset and progression of this cancer remains incompletely understood, with a number of different genetic, metabolic, and cellular factors all having been shown to contribute to this disease [4]. Given that RCC is linked to high rates of morbidity and mortality, it is essential that the etiology of this disease be better understood, and that novel disease-related diagnostic, prognostic, and/or therapeutic biomarkers be identified.

As is the case for other solid tumors, ccRCC develops in a stepwise manner over time, with initial genetic aberrations resulting in increasingly pathological changes in cell phenotype that eventually result in oncogenic transformation [5]. Many studies to date have employed an RNA sequencing (RNA-Seq) approach to survey cancer-related changes in gene expression at the whole genome level, yielding comprehensive and complex datasets [6]. By conducting systematic and integrative analyses of the relationship between differentially expressed genes and differentially engaged signaling pathways in tumor samples, it is possible to gain novel insights into ccRCC progression and therapeutic sensitivity. As such, leveraging currently available RNA-seq datasets can serve as a powerful tool for identifying better biomarkers of ccRCC that may help to guide its diagnosis or to better plan treatment in affected patients. At present, targeted therapies for ccRCC are limited due to a relatively poor understanding of which genes function as key drivers of disease [7]. Given that RNA-seq can aid in the identification of such key genes, further comprehensive transcriptomic analyses are essential in order to fully understand this complex and deadly disease.

In the present study, we conducted an integrative analysis of three previously published Gene Expression Omnibus (GEO) datasets (GSE66272, GSE100666, and GSE105261), and we utilized the ‘limma’ R package designed by the Bioconductor project [8] as well as a Venn diagram program in order to identify cancer-related differentially expressed genes (DEGs) shared among these three datasets. We then evaluated the enrichment of these DEGs in specific gene ontology (GO) annotated categories and in defined KEGG pathways using the DAVID tool. We further constructed a protein-protein interaction (PPI) network incorporating these DEGs, and we analyzed this network with the MCODE (Molecular Complex Detection) algorithm as a means of identifying significant gene modules therein. The top 20 hub genes within this network were then screened using cytoHubba, and were imported into the Kaplan Meier plotter database to evaluate their prognostic relevance. We further confirmed the ccRCC-related differential expression of key hub genes by evaluating their expression levels in ccRCC and normal kidney tissue samples in the GEPIA database. Through these approaches, we identified Tissue Inhibitor of Metalloproteinases 1 (*TIMP1*) as a gene potentially associated with ccRCC patient prognosis. Together, our results offer novel insights into the mechanistic basis for ccRCC development, and may suggest novel diagnostic and/or prognostic biomarkers that may be valuable therapeutic targets for the future treatment of ccRCC patients.

## RESULTS

### Identification of DEGs in renal cell carcinoma

**Figure 1:**
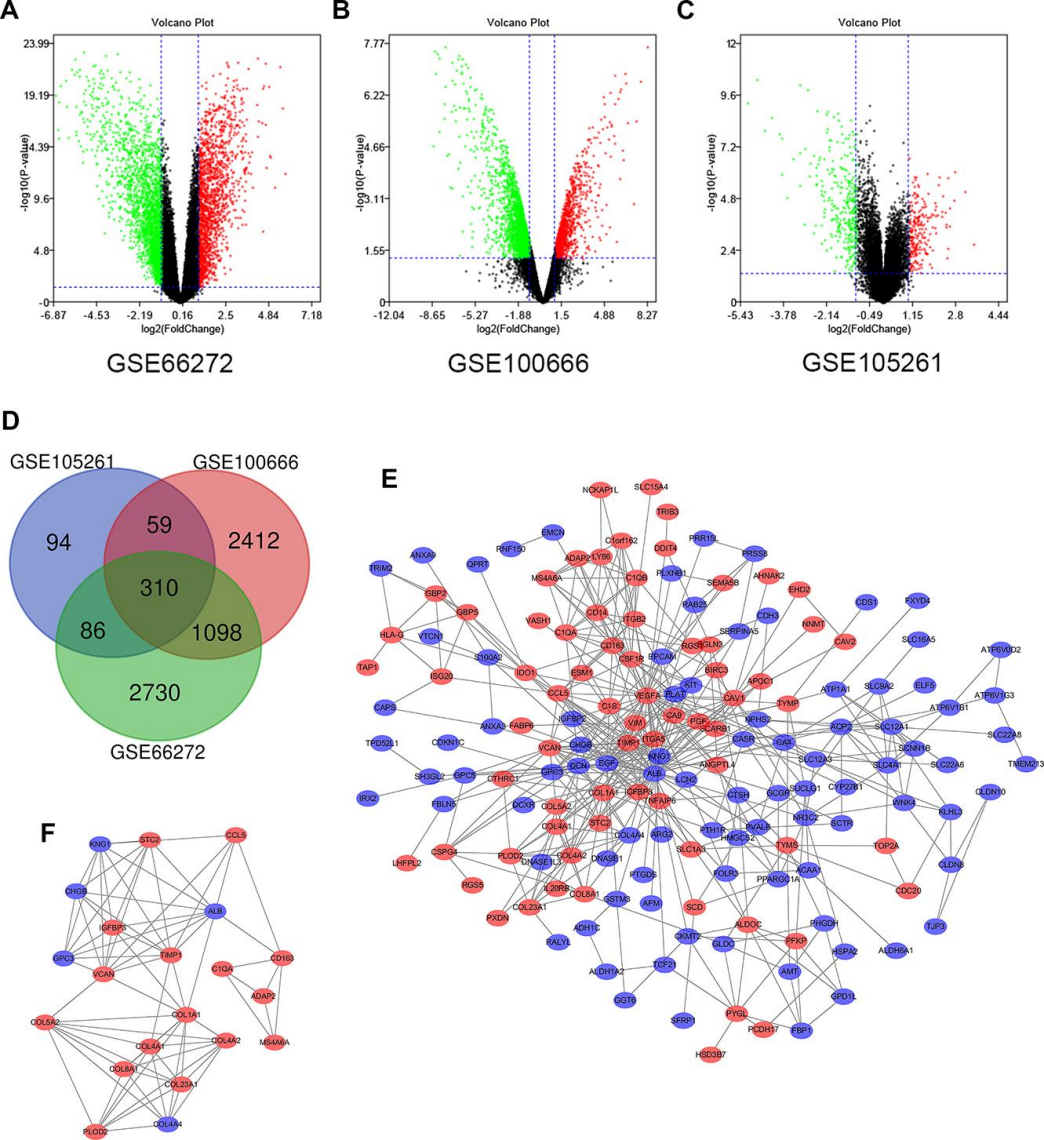
**Differential expression of genes in the two sets of samples, Venn diagram, PPI network and the most significant DEG module.** (**A**) GSE66272 data, (**B**) GSE100666 data, and (**C**) GSE105261 data. Red points represent upregulated genes screened on the basis of fold change > 1.0 and a corrected P-value of < 0.05. Green points represent downregulation of gene expression screened on the basis of fold change < 1.0 and a corrected P-value of < 0.05. Black points represent genes with no significant difference. (**D**) DEGs were selected with a |fold change| >1 and P-value <0.05 among the mRNA expression profiling sets GSE66272, GSE100666 and GSE105261. The 3 datasets exhibited an overlap of 310 genes. (**E**) The PPI network of DEGs was constructed using Cytoscape. (**F**) The most significant module was obtained from a PPI network with 21 nodes and 74 edges. Upregulated genes are marked in light red; downregulated genes are marked in light blue. Abbreviations: FC: fold change; GEO: Gene Expression Omnibus; DEGs; differentially expressed genes; PPI: protein–protein interaction.

In this study, 39 ccRCC samples and 39 paracancerous normal kidney tissue samples were analyzed. Using the limma software package, 4224, 3879 and 549 DEGs were extracted from GSE66272, GSE100666 and GSE105261, respectively. Figure 1A–1C show the DEGs in two sets of sample data from each of the three microarrays. Then use Venn diagram software to identify the DEGs common to these three datasets. A total of 310 common DEGs were detected in the ccRCC organization, including 177 genes were down-regulated (logFC <1) and 133 genes were up-regulated (logFC> 1) (Figure 1D).

### Gene ontology of DEGs and KEGG pathway analysis in renal cell carcinoma

In order to analyze the biological classification of DEGs, DAVID was used for functional and pathway enrichment analysis. GO analysis showed that BP changes in DEGs were significantly enriched in extracellular matrix organization, excretion, response to drugs, angiogenesis and response to hypoxia. The changes of CC were mainly concentrated in exosome, extracellular region, apical plasma membrane, extracellular space, and basolateral plasma membrane. The changes of MF mainly focus on protein homodimerization activity, ATPase binding, the same protein binding, extracellular matrix binding and extracellular matrix structure composition. Analysis of the KEGG pathway suggested that the DEGs were significantly enriched in carbon metabolism, biosynthesis of antibiotics, aldosterone-regulated sodium reabsorption, collecting duct acid secretion and phagosome (Table 1).

**Table 1:** GO and KEGG pathway enrichment analysis of DEGs in ccRCC samples.

**Term**	**Description**	**Count in gene set**	**P-value**
Upregulated			
GO:0030198	extracellular matrix organization	16	1.75E-11
GO:0001666	response to hypoxia	11	6.29E-07
GO:0001525	angiogenesis	12	8.72E-07
GO:0050900	leukocyte migration	8	3.53E-05
GO:0035987	endodermal cell differentiation	5	4.50E-05
GO:0005576	extracellular region	37	5.31E-10
GO:0031012	extracellular matrix	14	2.41E-07
GO:0005581	collagen trimer	9	3.42E-07
GO:0005201	extracellular matrix structural constituent	7	9.63E-06
GO:0042803	protein homodimerization activity	18	2.44E-05
hsa04510	Focal adhesion	12	1.38E-05
hsa04512	ECM-receptor interaction	7	3.70E-04
Downregulated			
GO:0007588	excretion	9	1.92E-09
GO:0034220	ion transmembrane transport	11	3.75E-05
GO:0001657	ureteric bud development	5	4.77E-04
GO:0035725	sodium ion transmembrane transport	6	6.95E-04
GO:0070062	extracellular exosome	81	7.51E-23
GO:0016324	apical plasma membrane	23	3.59E-14
GO:0016323	basolateral plasma membrane	15	1.37E-09
GO:0005887	integral component of plasma membrane	30	4.19E-05
GO:0005272	sodium channel activity	4	3.50E-04
GO:0030506	ankyrin binding	4	8.46E-04
hsa04960	Aldosterone-regulated sodium reabsorption	7	6.54E-06
hsa04966	Collecting duct acid secretion	6	1.57E-05
hsa01130	Biosynthesis of antibiotics	12	4.73E-05
hsa01200	Carbon metabolism	9	6.38E-05

### Modular analysis via DEGs protein-protein interaction (PPI) network

To determine the modular structure, STRING online database (available online: http://string-db.org) and the Cytoscape software were used the combined 310 DEGs to construct the PPI network of DEGs (Figure 1E) with the most important module obtained by using Cytoscape (Figure 1F). The functional analyses of genes involved in this module were analyzed using DAVID. The results of GO analysis showed that the changes of BPs of important module genes were significantly rich in collagen catabolic process, extracellular matrix organization, response to peptide hormone, cellular response to amino acid stimulus and platelet degranulation (Table 2). The CC changes of important module genes are mostly concentrated in extracellular region, collagen trimer, endoplasmic reticulum lumen, extracellular matrix, and collagen type IV trimer (Table 2). The MF changes of important module genes are mainly concentrated on extracellular matrix structural constituent, platelet-derived growth factor binding and protein binding (Table 2). KEGG pathway analysis showed that important modular genes were mostly enriched in receptor interaction, protein digestion and absorption, PI3K-Akt signaling pathway, focal adhesion and amoebiasis (Table 2).

**Table 2:** GO and KEGG pathway enrichment analysis of the significant module in ccRCC samples.

**Term**	**Description**	**Count in gene set**	**P-value**
Gene Ontology			
GO:0030574	collagen catabolic process	6	4.10E-09
GO:0030198	extracellular matrix organization	7	2.60E-08
GO:0043434	response to peptide hormone	3	8.90E-04
GO:0005576	extracellular region	16	1.48E-12
GO:0005581	collagen trimer	6	4.29E-08
GO:0005788	endoplasmic reticulum lumen	7	4.34E-08
GO:0031012	extracellular matrix	6	1.39E-05
GO:0005201	extracellular matrix structural constituent	6	9.42E-09
Biological pathway			
hsa04512	ECM-receptor interaction	5	1.09E-05
hsa04974	Protein digestion and absorption	5	1.14E-05
hsa05146	Amoebiasis	5	2.40E-05
hsa04510	Focal adhesion	5	3.20E-04

### Hub gene selection and survival analysis based on TCGA

**Figure 2:**
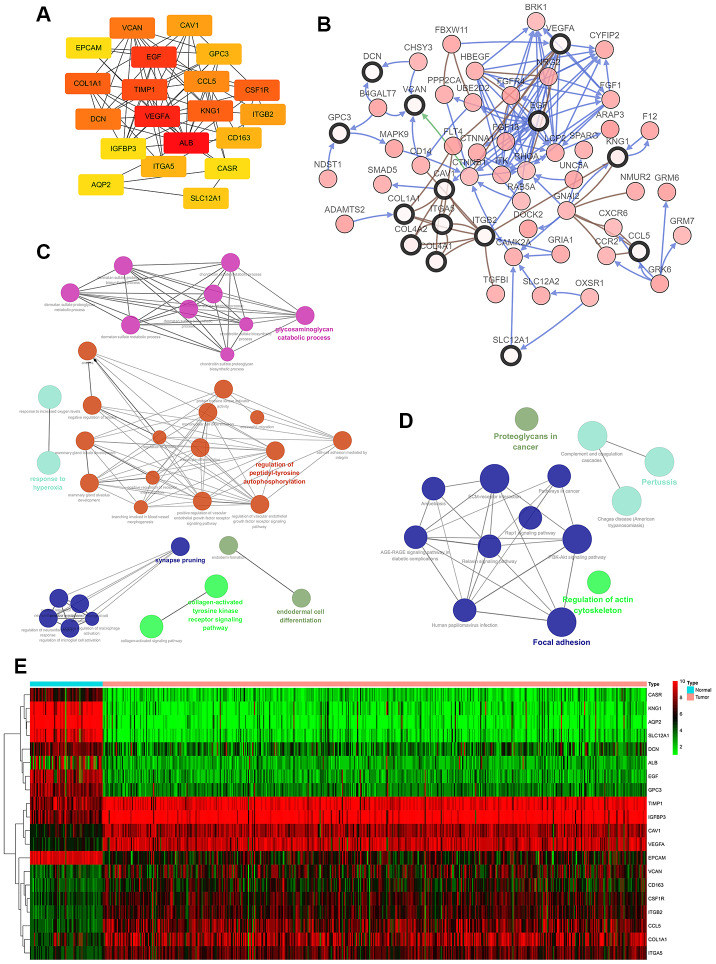
**Interaction network and analysis of hub genes.** (**A**) The 20 most important hub genes were screened using the Cytoscape software plugin cytoHubba. (**B**) Hub genes and their co-expressed genes were analyzed using the cBioPortal. Nodes with a bold black outline represent hub genes. Nodes with thin black outlines represent co-expressed genes. (**C**) Biological processes functional annotation analysis of hub genes was performed using ClueGO and CluePedia. Different colors of nodes refer to the functional annotation of ontologies. Corrected P value <0.01 was considered statistically significant. (**D**) KEGG functional annotation analysis of hub genes was performed by ClueGO and CluePedia. Different colors of nodes refer to the functional annotation of ontologies. Corrected P value <0.01 was considered statistically significant. (**E**) Hierarchical clustering heatmap of the 20 most important hub genes was constructed from a TCGA cohort. Red indicates that the relative expression of genes was upregulated, green indicates downregulation, and black indicates that no significant change in gene expression was observed; gray indicates that signal strength was not high enough to be detected. Abbreviation: TCGA: the cancer genome atlas program; KEGG: Kyoto Encyclopedia of Genes and Genomes.

30 hub genes were generated through filtering according to the criterion of degree of connectivity >10 (each node had more than 10 interactions). The 20 genes with the most significant degree of connectivity were *VCNA, CAV1, EPCAM, EGF, GPC3, COL1A1, TIMP1, CCL5, CSF1R, DCN, VEGFA, KNG1, ITGB2, IGFBP3, ALB, CD163, ITGA5, CASR, AQP2, SLC12A1* (Figure 2A). Table 3 shows the names, abbreviations and functions of these genes. The cBioPortal online platform was used to analyze the network of hub gene and their co-expressed genes (Figure 2B). The bioprocess analysis of central genes and the enrichment analysis of the Kyoto Encyclopedia of Genes and Genomes (KEGG) are shown in Figures 2C, 2D. Hierarchical clustering indicated that analysis of hub genes essentially allowed kidney cancer samples to be distinguished from non-cancer samples (Figure 2E).

**Table 3:** Functional roles of 20 hub genes.

**No.**	**Gene symbol**	**Full name**	**Function**
1	VCAN	Versican	Pathways: chondroitin sulfate/dermatan sulfate metabolism and diseases of glycosylation. GO: calcium ion binding and extracellular matrix structural constituent
2	CAV1	Caveolin 1	Pathways: Focal Adhesion and TNF signaling (REACTOME). GO: identical protein binding and signaling receptor binding.
3	EPCAM	Epithelial Cell Adhesion Molecule	Pathways: Cell surface interactions at the vascular wall and Embryonic and Induced Pluripotent Stem Cell Differentiation Pathways and Lineage-specific Markers
4	EGF	Epidermal Growth Factor	Pathways: Gastric cancer and Vesicle-mediated transport. GO: calcium ion binding and epidermal growth factor receptor binding.
5.	GPC3	Glypican 3	Pathways: Chondroitin sulfate/dermatan sulfate metabolism and Metabolism of fat-soluble vitamins. GO: heparan sulfate proteoglycan binding and peptidyl-dipeptidase inhibitor activity.
6	CCL5	C-C Motif Chemokine Ligand 5	Pathways: PEDF Induced Signaling and Innate Immune System. GO: protein homodimerization activity and chemokine activity.
7	CSF1R	Colony Stimulating Factor 1 Receptor	Pathways: GPCR Pathway and Nanog in Mammalian ESC Pluripotency. GO: protein homodimerization activity and protein kinase activity.
8	TIMP1	TIMP Metallopeptidase Inhibitor 1	Pathways: GPCR Pathway and Matrix Metalloproteinases. GO: cytokine activity and protease binding.
9	COL1A1	Collagen Type I Alpha 1 Chain	Pathways: IL4-mediated signaling events and Integrin Pathway. GO: identical protein binding and platelet-derived growth factor binding.
10	DCN	Decorin	Pathways: Chondroitin sulfate/dermatan sulfate metabolism and Diseases of glycosylation. GO: collagen binding.
11	VEGFA	Vascular Endothelial Growth Factor A	Pathways: VEGF Signaling Pathway and Bladder cancer. GO: protein homodimerization activity and protein heterodimerization activity.
12	KNG1	Kininogen 1	Pathways: Collagen chain trimerization and amb2 Integrin signaling. GO: signaling receptor binding and cysteine-type endopeptidase inhibitor activity.
13	ITGB1	Integrin Subunit Beta 2	Pathways: Activated TLR4 signalling and Focal Adhesion. GO: protein heterodimerization activity.
14	IGFBP3	Insulin Like Growth Factor Binding Protein 3	Pathways: TP53 Regulates Transcription of Cell Death Genes and Celecoxib Pathway, Pharmacodynamics. GO: fibronectin binding and insulin-like growth factor I binding.
15	ALB	Albumin	Pathways: Defective SLCO1B1 causes hyperbilirubinemia, Rotor type (HBLRR) and Synthesis of bile acids and bile salts. GO: enzyme binding and chaperone binding.
16	CD163	CD163 Molecule	Pathways: Binding and Uptake of Ligands by Scavenger Receptors and Dendritic Cells Developmental Lineage Pathway. GO: scavenger receptor activity.
17	CASR Calcium Sensing Receptor	Pathways: Parathyroid hormone synthesis, secretion and action and E-cadherin signaling in keratinocytes. GO: G protein-coupled receptor activity and protein kinase binding.
18	ITGA5	Integrin Subunit Alpha 5	Pathways: Cell surface interactions at the vascular wall and Focal Adhesion. GO: integrin binding and epidermal growth factor receptor binding.
19	AQP2	Aquaporin 2	Pathways: Aquaporin-mediated transport and Glucose / Energy Metabolism. GO: actin binding and PDZ domain binding.
20	SLC12A1	Solute Carrier Family 12 Member 1	Pathways: Neuroscience and Diuretics Pathway, Pharmacodynamics. GO: transporter activity and sodium:potassium:chloride symporter activity.

**Figure 3:**
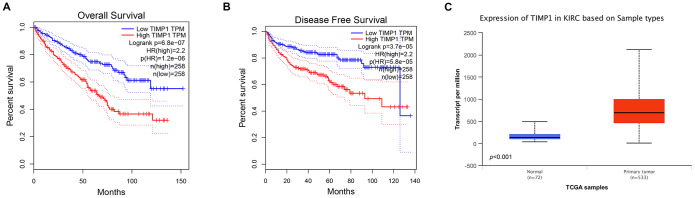
**Univariate survival analysis of hub genes was performed using Kaplan-Meier curves.** TIMP1 showed significant differences in both OS (**A**) and DFS (**B**) in ccRCC samples (Logrank P < 0.05). (**C**) Transcriptional levels of TIMP1 expression were found expressed in 533 ccRCC tissues compared with 72 normal tissues (p<0.0001). Abbreviation: DFS: disease-free survival; OS: overall survival; ccRCC: clear cell renal cell carcinoma.

In order to determine whether the pivotal genes in Clear cell renal cell carcinoma had clinical relevance, correlation analysis was performed with clinical correlation to kidney cancer outcomes according to the report of the Cancer Genome Atlas (TCGA) on the renal cancer data set. Using data from GEPIA, it was noted that ccRCC patients exhibiting genomic alteration in *TIMP1* demonstrated a reduction in overall and disease-free survival (P=6.8x10^-7^ for overall survival and P=3.7 x10^-5^ for disease-free survival) (Figure 3A, 3B). Therefore, it was considered that the hub gene *TIMP1* may play a key role in the progression of clear cell renal cell carcinoma.

### Analysis of the most significant hub gene

**Figure 4:**
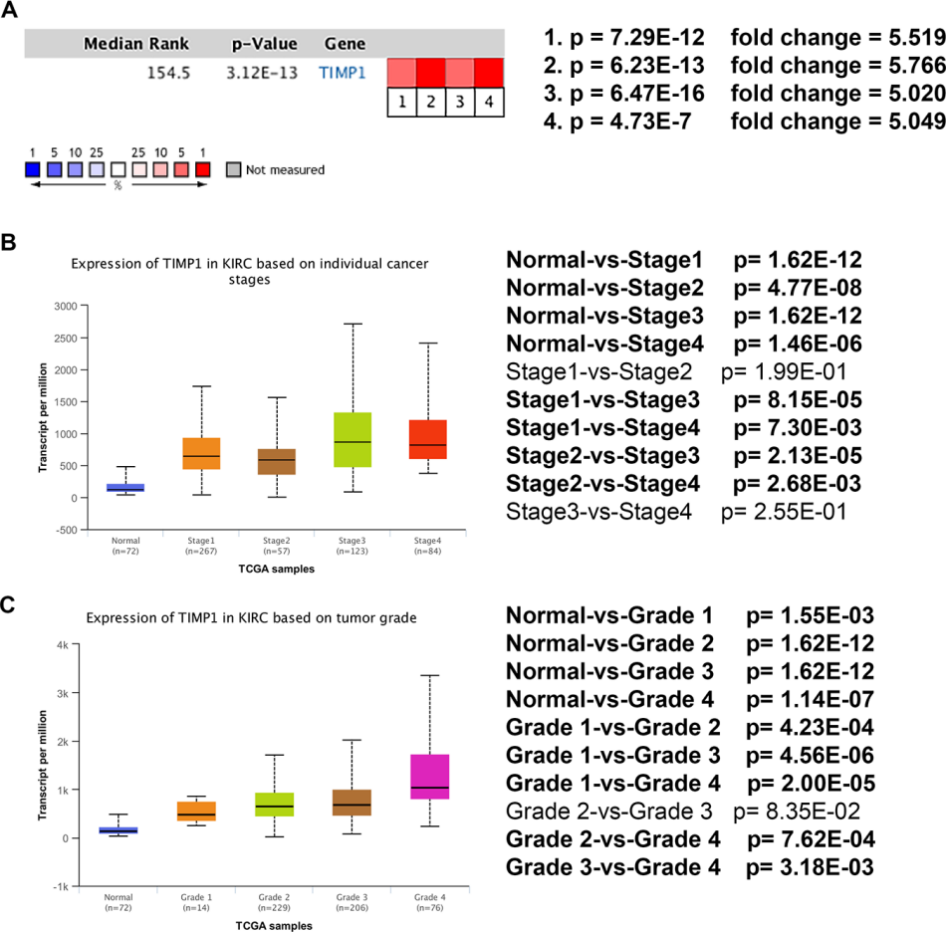
**Transcriptional expression of *TIMP1* significantly correlated with advanced clinicopathological parameters and poor survival outcomes in ccRCC patients.** (**A**) Oncomine analysis of cancer vs. normal tissue of *TIMP1*. Heat maps of *TIMP1* gene expression in clinical hepatocellular carcinoma samples vs. normal tissues. 1. Clear Cell Renal Cell Carcinoma vs. Normal, Higgins, Am J Pathol, 2003 [49]. 2. Clear Cell Renal Cell Carcinoma vs. Normal, Yusenko, BMC Cancer, 2009 [50]. 3. Clear Cell Renal Cell Carcinoma vs. Normal, Jones J, Clin Cancer Res, 2005 [51]. 4. Clear Cell Renal Cell Carcinoma vs. Normal, Gumz ML, Clin Cancer Res, 2007 [52]. (**B**) Transcriptional expression of *TIMP1* was significantly correlated with AJCC stage, patients who were in a more advanced stage tended to express higher mRNA expression of *TIMP1*. (**C**) Transcriptional expression of *TIMP1* was significantly correlated with ISUP grade. Patients in a more advanced grade tended to exhibit elevated *TIMP1*mRNA expression. Abbreviations: ccRCC: clear cell renal cell carcinoma.

Based on RNA sequence data from the TCGA database, TIMP1 mRNA expression was compared between kidney tumor samples and adjacent normal tissues. Transcriptional level data demonstrated that *TIMP1* expression was highly in 533 ccRCC tissues compared with 72 normal tissues (Figure 3C). Consistent with this finding, Oncomine analysis of cancerous and normal tissues showed that TIMP1 was greatly overexpressed in ccRCC samples from different data sets (Figure 4A). As shown in Figure 4B, *TIMP1* mRNA expression in ccRCC samples was significantly correlated with late clinical stage, and the highest *TIMP1* mRNA expression was observed in stages 3 and 4. Similarly, the relationship between *TIMP1* mRNA expression and different pathological grade was determined, which suggested that *TIMP1* mRNA expression was significantly correlated with pathological grade (Figure 4C). In general, increased expression of TIMP1 mRNA in ccRCC patients was significantly correlated with advanced clinicopathologic parameters.

**Figure 5:**
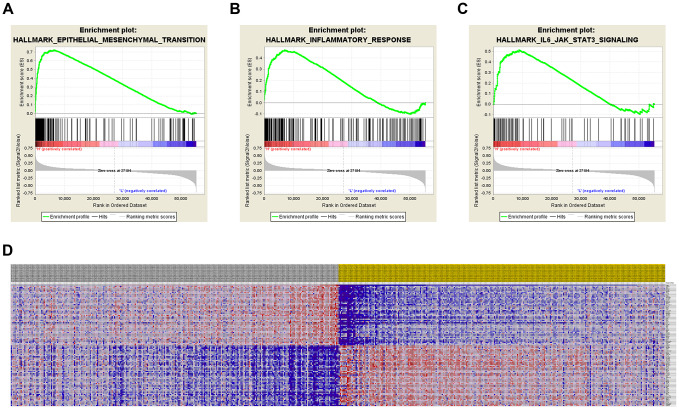
**Significantly related genes and hallmarks pathways in ccRCC obtained by GSEA.** GSEA was used to perform hallmark analysis for *TIMP1* (**A**–**C**). The most significant pathways included epithelial-mesenchymal-transition, inflammatory-response, and IL6/JAK/STAT3 signaling. (**D**) Transcriptional expression profiles of the 100 most significant genes expressed as a heat map.

GSEA was used for hallmark analysis of *TIMP1*. The results suggest that the most significantly involved pathways included epithelial-mesenchymal-transition, inflammatory-response and IL6/JAK/STAT3 signaling, as displayed in Figures 5A–5C. In addition, the expression profiles of the 100 most significant genes are displayed in a heat map (Figure 5D).

### TIMP1 expression correlates with interstitial phenotype in ccRCC cells

**Figure 6:**
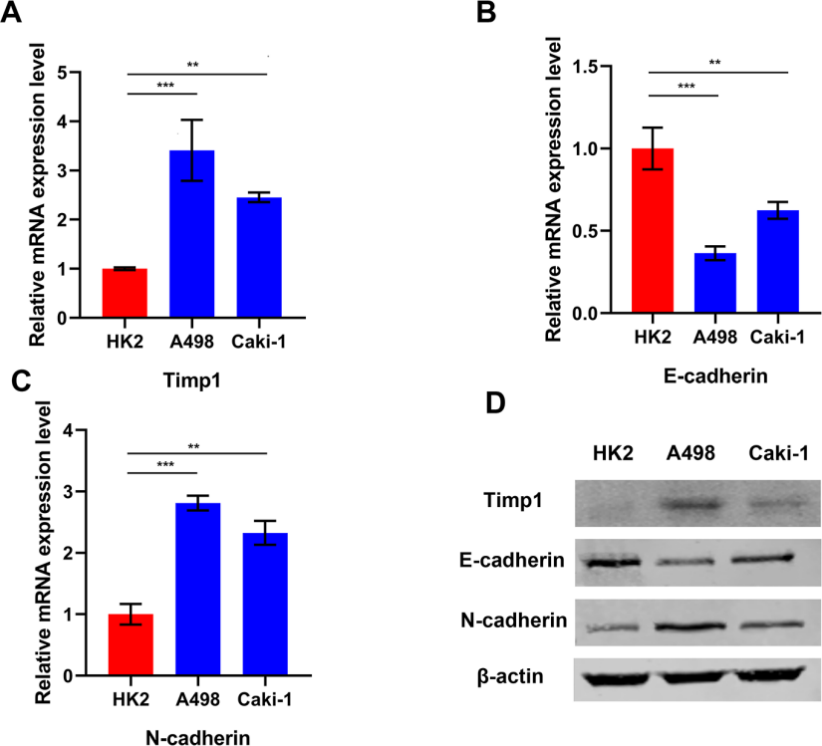
**TIMP1 expression was associated with mesenchymal phenotype in ccRCC cell lines.** Relative mRNA expression of TIMP1 (**A**), E-cadherin (**B**) and N-cadherin (**C**) in 2 ccRCC cell lines and a normal cell line presented separately as histograms. (**D**) TIMP1, E-cadherin, and N-cadherin protein levels as determined by Western blot analysis. β-actin was used as the internal control. (* p<0.05, ** p<0.01, *** p<0.001 by t-test).

GSEA enrichment analysis indicated that TIMP1 was involved in the EMT process. In order to understand the relationship between *TIMP1* and the mesenchymal phenotype in ccRCC cells, TIMP1 and EMT represent markers (such as E-cadherin and N-) in 2 different ccRCC cell lines (A498 and Caki-1) and HK2 cells (normal renal cell lines) were examined by qRT-PCR and Western blot (Figure 6A–6D). As consistently shown by qRT-PCR and Western blot analysis, *TIMP1* was abundantly expressed in A498 and Caki-1 mesenchymal cells, which exhibit higher N-cadherin and lower E-cadherin expression than HK2 cells, indicating that that *TIMP1* level may be related to the mesenchymal phenotype of ccRCC cell line.

To demonstrate the correlation between mesenchymal genes and *TIMP1* expression, 200 about mesenchymal genes in GSEA Molecular Signatures Database HALLMARK EPITHELIAL MESENCHYMAL TRANSITION gene set (For more details, please refer to the follow website link: https://www.gsea-msigdb.org/gsea/msigdb/cards/HALLMARK_EPITHELIAL_MESENCHYMAL_TRANSITION.html) were first retrieved. Next, Pearson and Spearman correlation test were carried out to calculate the correlation and P value between mesenchymal genes and *TIMP1* expression within 3 datasets (GSE66727, GSE100666, GSE105261). The results were shown in Supplementary Table 1. Totally, expression values of 190/200 genes were found in three datasets and all genes showed significant correlation with *TIMP1*.

### Knockdown of TIMP1 induces MET of A498 and Caki-1 cells

**Figure 7:**
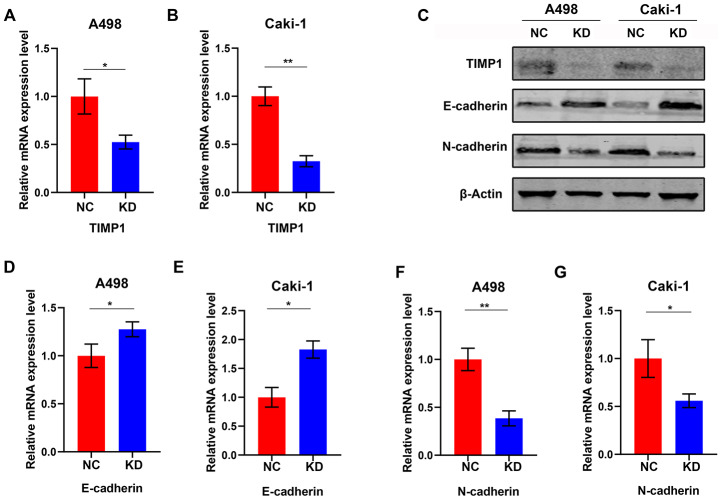
**Knockdown of TIMP1 induced MET in A498 and Caki-1 cells.** qRT-PCR (**A**, **B**) analysis of TIMP1 expression in A498 and Caki-1 cells transfected with TIMP1 shRNA (KD) and normal controls (NC). (**C**) Western blot analysis of TIMP1 expression in A498 and Caki-1 cells transfected with TIMP1 shRNA (KD) from a normal control (NC). Expression levels of protein (**C**) and mRNA (**D**–**G**) of the EMT markers are shown. (* p<0.05, ** p<0.01, *** p<0.001 by t-test).

To study the effect of TIMP1 knockdown on EMT in ccRCC cells, lentiviruses with TIMP1 shRNA or NC were transfected into A498 and Caki-1 cells. Stably passaged A498-KD, A498-NC, Caki-1-KD and Caki-1-NC cells were thus obtained. qRT-PCR demonstrated a dramatic decrease in *TIMP1* mRNA expression in A498 and Caki-1 cells following lentivirus transfection (Figure 7A, 7B). Western blot analysis confirmed that TIMP1 protein was inhibited in both A498 and Caki-1 cells (Figure 7C). Through TIMP1 knockdown, Western blot analysis and qRT-PCR confirmed the enhanced expression of epithelial markers (e.g. E-cadherin) and interstitial markers (e.g. N- cadherin) expression is reduced in A498-KD and Caki-1-KD cells (Figure 7C–7G). These results suggest that the knockdown of *TIMP1* in A498 and Caki-1 cells induced transition to the epithelial phenotype.

### Knocking down TIMP1 inhibits migration and invasion of A498 and Caki-1 cells

**Figure 8:**
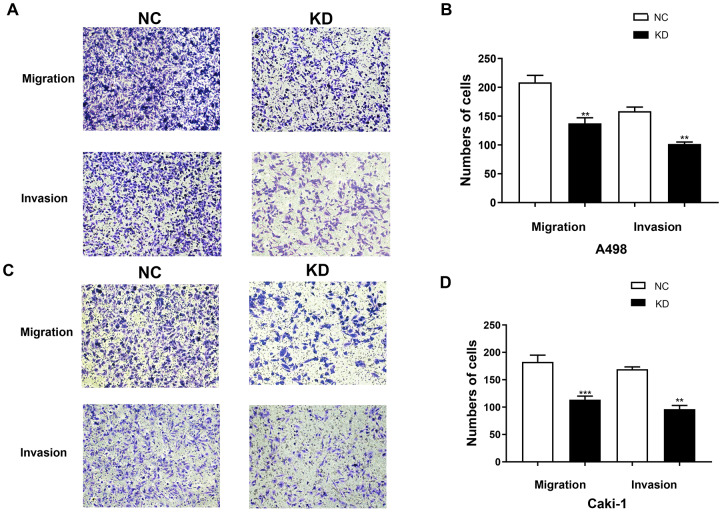
**TIMP1 knockdown inhibited the migration and invasion of A498 and Caki-1 cells.** (**A**) Migration and Matrigel invasion assays of A498 cells transfected with TIMP1 shRNA or NC were evaluated. (**C**) Migration and Matrigel invasion assays of Caki-1 cells transfected with TIMP1 shRNA or NC were evaluated. (**B**, **D**) Migrated and invaded cells were counted in 3 random 100× fields (* p<0.05, ** p<0.01, *** p<0.001 using a t-test).

The cell migration and invasion characteristics are considered to be important consequences of EMT [9, 10], so the effect of TIMP1 knockdown on the migration and invasiveness of A498 and Caki-1 cells was studied using transwell migration and invasion assays. The migration rate and invasion rate of A498-KD and Caki-1-KD cells decreased compared with A498-NC and Caki-1-NC cells, respectively (Figure 8A–8D).

## DISCUSSION

ccRCC is among the most common malignancies around the world. Early diagnosis and treatment of ccRCC can greatly improve prognosis and survival of patients with ccRCC. Although the underlying pathogenesis and molecular mechanisms of ccRCC development and progression have been studied using multiple omics technologies, the global mortality rate for ccRCC is still high over past decades. A series of studies reported that ccRCC is accompanied with accumulation of abnormal agents at cellular and molecular level, such as epigenetic, transcriptomic, miRNA, proteomic and metabolomic alternations [11–14]. These multiple omics studies have attempted to identify early diagnostic biomarkers of renal cancer, and highlight potential heterogeneity and molecular commonalities between different stages of renal cancer. It is well known that ccRCC exhibits important molecular heterogeneity, involving multiple changes of genetic and protein expression levels. Therefore, a series of appropriately-selected candidate biomarkers representative of all such tumors may be essential for identification. With the development of bioinformatic analysis, a number of molecules in ccRCC have been screened as potential novel prognostic biomarkers, but few of them has been systematically validated. Neither of them has been compared with each other to confirm additional studies that are required to identify candidate biomarkers. Potential biomarkers for early detection and prediction of ccRCC progression are still largely unknown.

In the present study, we conducted analysis of three raw microarray datasets, including 39 renal tumor samples and 39 adjacent normal kidney tissues. We screened out 310 DEGs from these datasets, which included 133 up-regulated and 177 down-regulated genes. GO was performed through DAVID, which indicated that these identified DEGs were mostly enriched in collagen catabolic processes, extracellular matrix organization, extracellular region, collagen trimer and extracellular matrix structural constituents. The functional relevance of the DEGs was evaluated by pathway enrichment based on the KEGG database. The PPI networks were constructed and visualized by using the STRING database and Cytoscape software. The 20 most significant hub genes were screened out including *VCNA, CAV1, EPCAM, EGF, GPC3, COL1A1, TIMP1, CCL5, CSF1R, DCN, VEGFA, KNG1, ITGB2, IGFBP3, ALB, CD163, ITGA5, CASR, AQP2, SLC12A1.* To screen for prognosis-related genes, we found that patients with significantly reduced OS and DFS also had highly expressed *TIMP1*.

*TIMP1* has been demonstrated to be involved in the progression of tumors involving lung adenocarcinoma, glioma, prostate cancer, breast cancer, colorectal cancer and a number of other cancers [15]. TIMP1 expression is often remarkably enhanced in the in the late stages of such tumors, in addition to in patients with endometrial, breast or brain cancer who have a shorter time prior to relapse [16–18]. Consistent with this, a lack of *TIMP1* immunostaining is related to improved prognosis in patients with lymph node-positive high-grade breast cancer [19]. In contrast, an increase in *TIMP1* levels in tumor tissues was linked to a remarkable declined overall survival in breast cancer patients receiving standard adjuvant chemotherapy [16]. While *TIMP1* has been shown to be overexpressed in many malignant tumors, the prognostic value and potential function of *TIMP1* in ccRCC is still unclear. In the present study, the effect of different expression levels of *TIMP1* in ccRCC on prognosis was explored. We found that the increased expression of *TIMP1* in ccRCC was positively correlated with malignant behavior, and high levels of *TIMP1* predicted high risk of recurrence and reduced overall survival. This observation reveals a novel role for *TIMP1* that influences ccRCC progression through potential DNA damage variants. Meanwhile, through functional enrichment and GSEA analysis, we found that *TIMP1* was enriched in several hallmark signaling pathways, including epithelial-mesenchymal transition (EMT), IL6-JAK-STAT3 signaling pathway, and inflammatory response in ccRCC samples.

Inflammation is observed as a basic biological behavior and confirmed as a hallmark of many malignant tumors [20]. Cancer-associated inflammation involves crosstalk between malignant and non-malignant cells in an autocrine and paracrine fashion through mediators such as cytokines, chemokines and prostaglandins [21]. With the combined force of genetic alteration, inflammatory tumor microenvironments contribute towards tumor growth and distant metastasis [22]. Furthermore, the carcinogenic effect of inflammatory response could be inhibited by using anti-inflammatory drugs [23]. For instance, treatment with the anti-inflammatory drug dexamethasone significantly inhibits malignant transformation by inhibiting EMT, the process epithelial cells undergo to gain migration and invasion capabilities [24]. In addition, tumor microenvironments consist of multiple different inflammatory cells and mediators. The development of inhibitors targeting these risk factors markedly delays the development and metastasis of tumors [21]. Therefore, learning more about the pro-inflammatory mechanisms of *TIMP1* may provide promise strategy for treatment of ccRCC.

Studies have demonstrated that *TIMP1* may be involved in promotion of progression of colorectal cancer, possibly serving as a prognostic hallmark for colorectal cancer [25, 26]. In addition, due to the difficulty in diagnosing pancreatic cancer, studies have demonstrated that *TIMP1* may serve as an early diagnostic marker for pancreatic cancer.

The expression of *TIMP1* in renal cell carcinoma regulates the IL6-JAK-STAT3 pathway. Previous studies have illustrated that IL-6 was related to tumorigenesis and EMT of non-small cell lung cancer [27], distant dissemination of prostate cancer and breast cancer [28], regeneration and drug resistance of breast cancer stem cells [29]. Within tumor microenvironments, IL-6/JAK/STAT3 signaling contributes to growth and metastasis of tumor cells by inhibiting the anti-tumor immune response [30]. These findings suggest that suppression of IL-6 pathway would be a novel strategy for treatment of refractory tumors.

Multiple studies have demonstrated that EMT had pre-metastatic effect in progression of tumor cells [31, 32]. EMT is an embryonic program that relaxes cell-cell adhesion complexes and confers elevated migration and invasion capabilities. After undergoing EMT, Cancer cells are more aggressive and invasive. Moreover, these cells obtain stem-like characteristics, and are resistance to apoptosis [33]. Research data indicates that the EMT program can also facilitate cancer cells to produce pro-inflammatory factors [34], and the inflammation in turn promotes EMT program in tumors [34]. Therefore, these two phenomena may form positive feedback in the first steps of tumor formation and transfer alliance. Consistent with this, subcutaneous injecting gastric quiescent cancer stem cells to nude mice would induce EMT-like changes and form larger xenografts [35]. In addition, primary tumors may also be resistant to treatment due to the EMT/inflammation alliance [36]. GSEA analysis has shown that elevated expression of *TIMP1* is involved in the biological processes of EMT, which may explain why enhanced expression of *TIMP1* is correlated with declined overall survival of ccRCC patients. Future studies should now focus on the mechanism by which *TIMP1* regulates the biological process of EMT, perhaps providing a target for the treatment of kidney cancer.

The present study attempted to identify candidate DEGs in primary ccRCC and *TIMP1* was screened out. Furthermore, we demonstrated that overexpression of *TIMP1* may be associated with invasion and migration of ccRCC cells. However, the underlying mechanisms of *TIMP1* pathway in ccRCC was failed to investigate in this study. Therefore, more research in future should explore the detailed mechanisms of hub genes in progression of ccRCC.

## CONCLUSIONS

In conclusion, our study identified hub genes and genes expressed differentially in healthy compared with primary ccRCC samples from GEO datasets, which may provide novel biomarkers for diagnosis and treatment of ccRCC. In addition, the study is the first to reveal that *TIMP1* promotes progression of ccRCC and predicts poor prognosis of ccRCC patients. The study may provide novel and promising insights for subsequent research for elucidation of the molecular mechanisms of ccRCC. In this regard, large clinical cohort study and further elucidation are imperative to uncover the underlying mechanisms and pathogenesis for ccRCC patients.

## MATERIALS AND METHODS

### Gene expression profiling

We downloaded the publicly available GSE66272, GSE100666, and GSE105261 gene expression datasets from NCBI-GEO. These studies compared gene expression profiles in kidney tumor and normal kidney tissue samples. GSE66272 data were generated with the GPL570 platform (Affymetrix Human Genome U133 Plus 2.0 Array), and were based on 27 tumor and 27 normal tissue samples. GSE100666 data were generated with the GPL16951 platform (Phalanx Human OneArray Ver. 6 Release 1), and were based on 3 ccRCC and 3 normal tissue samples. GSE105261 data were generated with the GPL10558 platform (Illumina HumanHT-12 V4.0 expression BeadChip), and were based on analyses of 9 tumor and 9 paracancerous normal kidney tissue samples.

### Standardization and elucidation of DEGs

After downloading matrix files for each GEO dataset, we utilized a Robust Multi-Array Average (RMA) algorithm in order to adjust data for background signals, and to conduct quantile normalization and final oligonucleotides-per-transcript summarization with a median polish algorithm. The R Impute package was used to impute missing values based on a k-nearest neighbor (KNN) approach. Bayes methods were used to adjust for batch effects with the sva R package, as published previously, with R v3.6.1 being used for all analyses [37].

DEGs were identified via comparing ccRCC and normal kidney tissue gene expression profiles based on adjusted P-values, fold change values, and Benjamini and Hochberg FDR values. We removed any probe sets that did not correspond to a gene symbol, while genes with multiple probe sets were averaged. DEGs were considered to be significant if they met the criteria: |log2FC| >1.00, P <0.05. An online Venn diagram program was then used to identify shared DEGs among these three datasets, with data being uploaded in a TXT format. Genes were up- and down-regulated if logFC values were > 0 and < 0, respectively.

### Functional enrichment analysis

The DAVID (http://david.ncifcrf.gov) (version 6.7) database was used to conduct functional annotation and pathway enrichment analyses for identified DEGs [38]. The KEGG database was used for pathway enrichment analysis of these DEGs in an effort to gain high-level insights into their potential functional relevance [39]. GO enrichment analyses were conducted to assess the enrichment of these DEGs in specific biological processes, molecular functions, and cell components (BPs, MFs, and CCs, respectively) [40]. Herein, DAVID was utilized for these enrichment analyses.

### PPI network analysis

DEGs were grouped into a PPI network with the STRING database (http://string-db.org) [41], with a score of >0.4 being used as a significance cutoff for network construction. The network was then visualized and analyzed with the Cytoscape tool (v3.7.1) [42]. The Cytoscape Molecular Complex Detection (MCODE) plugin (v1.5.1) was further used for topological clustering within the network as a means of identifying significantly interconnected gene modules within the overall PPI network [43]. The following criteria were used for significant module identification: degrees of cut-off = 2, node score cut-off = 0.2, Max depth = 100, K-score = 2.

### Hub gene selection and analysis

Hub genes were identified as genes that had a ≥10 degree of connectivity. We then utilized cBioPortal (http://www.cbioportal.org) [44] to identify genes that were co-expressed with these hub genes. We additionally utilized the ClueGO Cytoscape plugin, which allows for the visualization of non-redundant terms associated with gene clusters in networks that have been grouped based on functionality [45]. We utilized ClueGO v2.5.4 and CluePedia v1.5.4 to identify and visualize biological processes associated with these hub genes [46]. We additionally performed hub gene hierarchical clustering.

### Survival analysis

Differences in survival between groups were evaluated via a Kaplan-Meier approach, with disease-free survival (DFS) as the primary endpoint. DFS was defined as the time between curative treatment and progression, death, or second-line treatment. As a secondary endpoint in these analyses, we assessed overall survival (OS) from diagnosis/treatment to death or most recent follow-up. After comparing survival between groups using Kaplan-Meier curves, 95% confidence intervals (95% CIs) were generated and curves were compared via log-rank tests. The sum of hub gene weights was used to determine overall scores.

### Data processing of Gene set enrichment analysis (GSEA)

A GSEA approach was used to analyze TCGA data with the Category v2.10.1 package. In individual analyses, Student’s t-tests were used to generate scores to assess consistent changes in DEG expression within pathways of interest. A 1000x permutation test was employed to identify pathways that were significantly altered, and the potential for false-positive result detection was controlled by correcting P-values using the Benjamini and Hochberg false discovery rate approach [47]. Genes were found to be significantly related when adj. P was < 0.01 and FDR < 0.25. R v3.6.1 was used for these statistical analyses.

### Cell culture

F-12, McCoy’s 5A, and DMEM were used to culture HK-2, Caki-1, and A498 cells, respectively. In all three cases, media was supplemented with 10% FBS, 2 mM L-glutamine, and penicillin/streptomycin. All cell culture was conducted in a humidified 37°C 5% CO2 incubator.

### Lentiviral preparation

HEK 293T cells (American Type Culture Collection, USA) were co-transfected with lentiviral vectors and packaging plasmids using Lipofectamine 3000 (Invitrogen, Shanghai, China) based on provided directions. Following a 48 h incubation, lentivirus-containing supernatants were harvested.

### Lentiviral transduction

A total of 3×105 ccRCC cells were added to 6 cm tissue culture dishes and were incubated overnight, after which 200 μl of an appropriate lentiviral supernatant and polybrene (8 μg/ml) were added to each well. Cells were then incubated at 37°C, and on day three the virus-containing media was removed. Cells were then rinsed using PBS, and puromycin-containing media (2 μg/ml) was added. After overnight incubation, a subset of cells were collected for western blotting in order to identify stably transduced cells for further experimental utilization. Infection of target cells with lentiviral particles.

### qRT-PCR

Trizol (Invitrogen) was then used to extract total cellular RNA, after which SYBR® Premix Ex Taq (TaKaRa) was used for qRT-PCR reactions based on previously published protocols [48]. Primers used were: TIMP1 F: 5’- ACCACCTTATACCAGCGTTATGA -3’; TIMP1 R: 5’- GGTGTAGACGAACCGGATGTC -3’. Β-actin was used to normalize gene expression, with the 2-ΔΔCt approach being used to compare gene expression levels.

### Western blotting

RIPA buffer was used to lyse harvested cells. Protein was then separated via SDS-PAGE, transferred to NC membranes (GE Health Care Life Science). Blots were blocked with 5% skim milk in TBST, followed by an overnight incubation with appropriate primary antibodies at 4°C. Antibodies were: anti-TIMP1 (Abcam, ab61224), and anti-Actin (Zen bioscience, 220529). Blots were then probed for 1 h with appropriate secondary antibodies (1:5000), and chemiluminescence was then used for protein visualization.

### Transwell assay

A 24-well Transwell system (Greiner Bio-one, Switzerland) was employed for assays examining ccRCC cell invasive activity. The upper surface of these Transwell inserts was first coated using Matrigel (BD Bioscience, USA), after which 200 uL of serum-free media containing 2×105 cells was added to the upper chamber. Next, 500 uL of media containing 10% FBS was added to the lower compartment, and cells were incubated for 48 h at 37°C. A swab was then used to carefully remove cells remaining on the upper surface, while cells that had migrated to the lower surface were treated with 100% methanol for fixation followed by 0.1% crystal violet staining. A total of five fields of view per insert were then visualized at random via microscope (Olympus, Japan; 200x), with the number of cells in each field being quantified.

### Ethics approval

The Ethics Committee of Qilu Hospital of Shandong University approved the study.

## Supplementary Material

Supplementary Table 1

## References

[1] Siegel RL, Miller KD, Jemal A. Cancer statistics, 2019. CA Cancer J Clin. 2019; 69:7–34. 10.3322/caac.21551 30620402

[2] Chen W, Zheng R, Baade PD, Zhang S, Zeng H, Bray F, Jemal A, Yu XQ, He J. Cancer statistics in China, 2015. CA Cancer J Clin. 2016; 66:115–32. 10.3322/caac.21338 26808342

[3] Baldewijns MM, van Vlodrop IJ, Schouten LJ, Soetekouw PM, de Bruïne AP, van Engeland M. Genetics and epigenetics of renal cell cancer. Biochim Biophys Acta. 2008; 1785:133–55. 10.1016/j.bbcan.2007.12.002 18187049

[4] Linehan WM, Ricketts CJ. The metabolic basis of kidney cancer. Semin Cancer Biol. 2013; 23:46–55. 10.1016/j.semcancer.2012.06.002 22705279 PMC3563773

[5] Li QK, Pavlovich CP, Zhang H, Kinsinger CR, Chan DW. Challenges and opportunities in the proteomic characterization of clear cell renal cell carcinoma (ccRCC): A critical step towards the personalized care of renal cancers. Semin Cancer Biol. 2019; 55:8–15. 10.1016/j.semcancer.2018.06.004 30055950 PMC6624650

[6] Maekawa S, Suzuki A, Sugano S, Suzuki Y. RNA sequencing: from sample preparation to analysis. Methods Mol Biol. 2014; 1164:51–65. 10.1007/978-1-4939-0805-9_6 24927835

[7] Lai C, Teng X. [Molecular biological foundation of targeted therapy for metastatic renal cell carcinoma]. Zhejiang Da Xue Xue Bao Yi Xue Ban. 2016; 45:91–97. 27045248 10.3785/j.issn.1008-9292.2016.01.15 PMC10397009

[8] Smyth GK, Michaud J, Scott HS. Use of within-array replicate spots for assessing differential expression in microarray experiments. Bioinformatics. 2005; 21:2067–75. 10.1093/bioinformatics/bti270 15657102

[9] Yilmaz M, Christofori G. EMT, the cytoskeleton, and cancer cell invasion. Cancer Metastasis Rev. 2009; 28:15–33. 10.1007/s10555-008-9169-0 19169796

[10] Polyak K, Weinberg RA. Transitions between epithelial and mesenchymal states: acquisition of malignant and stem cell traits. Nat Rev Cancer. 2009; 9:265–73. 10.1038/nrc2620 19262571

[11] Moch H, Cubilla AL, Humphrey PA, Reuter VE, Ulbright TM. The 2016 WHO Classification of Tumours of the Urinary System and Male Genital Organs-Part A: Renal, Penile, and Testicular Tumours. Eur Urol. 2016; 70:93–105. 10.1016/j.eururo.2016.02.029 26935559

[12] Delahunt B, Srigley JR. The evolving classification of renal cell neoplasia. Semin Diagn Pathol. 2015; 32:90–102. 10.1053/j.semdp.2015.02.002 25753529

[13] Cancer Genome Atlas Research Network. Comprehensive molecular characterization of clear cell renal cell carcinoma. Nature. 2013; 499:43–49. 10.1038/nature12222 23792563 PMC3771322

[14] Davis CF, Ricketts CJ, Wang M, Yang L, Cherniack AD, Shen H, Buhay C, Kang H, Kim SC, Fahey CC, Hacker KE, Bhanot G, Gordenin DA, et al, and The Cancer Genome Atlas Research Network. The somatic genomic landscape of chromophobe renal cell carcinoma. Cancer Cell. 2014; 26:319–30. 10.1016/j.ccr.2014.07.014 25155756 PMC4160352

[15] Jackson HW, Defamie V, Waterhouse P, Khokha R. TIMPs: versatile extracellular regulators in cancer. Nat Rev Cancer. 2017; 17:38–53. 10.1038/nrc.2016.115 27932800

[16] Dechaphunkul A, Phukaoloun M, Kanjanapradit K, Graham K, Ghosh S, Santos C, Mackey JR. Prognostic significance of tissue inhibitor of metalloproteinase-1 in breast cancer. Int J Breast Cancer. 2012; 2012:290854. 10.1155/2012/290854 22988515 PMC3440855

[17] Aaberg-Jessen C, Christensen K, Offenberg H, Bartels A, Dreehsen T, Hansen S, Schrøder HD, Brünner N, Kristensen BW. Low expression of tissue inhibitor of metalloproteinases-1 (TIMP-1) in glioblastoma predicts longer patient survival. J Neurooncol. 2009; 95:117–28. 10.1007/s11060-009-9910-8 19430729

[18] Honkavuori M, Talvensaari-Mattila A, Puistola U, Turpeenniemi-Hujanen T, Santala M. High serum TIMP-1 is associated with adverse prognosis in endometrial carcinoma. Anticancer Res. 2008; 28:2715–19. 19035300

[19] Kuvaja P, Talvensaari-Mattila A, Pääkkö P, Turpeenniemi-Hujanen T. The absence of immunoreactivity for tissue inhibitor of metalloproteinase-1 (TIMP-1), but not for TIMP-2, protein is associated with a favorable prognosis in aggressive breast carcinoma. Oncology. 2005; 68:196–203. 10.1159/000086774 16006757

[20] Hanahan D, Weinberg RA. Hallmarks of cancer: the next generation. Cell. 2011; 144:646–74. 10.1016/j.cell.2011.02.013 21376230

[21] Crusz SM, Balkwill FR. Inflammation and cancer: advances and new agents. Nat Rev Clin Oncol. 2015; 12:584–96. 10.1038/nrclinonc.2015.105 26122183

[22] Singh R, Mishra MK, Aggarwal H. Inflammation, Immunity, and Cancer. Mediators Inflamm. 2017; 2017:6027305. 10.1155/2017/6027305 29234189 PMC5695028

[23] Pribluda A, Elyada E, Wiener Z, Hamza H, Goldstein RE, Biton M, Burstain I, Morgenstern Y, Brachya G, Billauer H, Biton S, Snir-Alkalay I, Vucic D, et al. A senescence-inflammatory switch from cancer-inhibitory to cancer-promoting mechanism. Cancer Cell. 2013; 24:242–56. 10.1016/j.ccr.2013.06.005 23890787

[24] Rhim AD, Mirek ET, Aiello NM, Maitra A, Bailey JM, McAllister F, Reichert M, Beatty GL, Rustgi AK, Vonderheide RH, Leach SD, Stanger BZ. EMT and dissemination precede pancreatic tumor formation. Cell. 2012; 148:349–61. 10.1016/j.cell.2011.11.025 22265420 PMC3266542

[25] Song G, Xu S, Zhang H, Wang Y, Xiao C, Jiang T, Wu L, Zhang T, Sun X, Zhong L, Zhou C, Wang Z, Peng Z, et al. TIMP1 is a prognostic marker for the progression and metastasis of colon cancer through FAK-PI3K/AKT and MAPK pathway. J Exp Clin Cancer Res. 2016; 35:148. 10.1186/s13046-016-0427-7 27644693 PMC5028967

[26] Huang R, Wang K, Gao L, Gao W. *TIMP1* Is A Potential Key Gene Associated with The Pathogenesis And Prognosis Of Ulcerative Colitis-Associated Colorectal Cancer. Onco Targets Ther. 2019; 12:8895–904. 10.2147/OTT.S222608 31802901 PMC6826183

[27] Lee SO, Yang X, Duan S, Tsai Y, Strojny LR, Keng P, Chen Y. IL-6 promotes growth and epithelial-mesenchymal transition of CD133+ cells of non-small cell lung cancer. Oncotarget. 2016; 7:6626–38. 10.18632/oncotarget.6570 26675547 PMC4872738

[28] Albino D, Civenni G, Rossi S, Mitra A, Catapano CV, Carbone GM. The ETS factor ESE3/EHF represses IL-6 preventing STAT3 activation and expansion of the prostate cancer stem-like compartment. Oncotarget. 2016; 7:76756–68. 10.18632/oncotarget.12525 27732936 PMC5363547

[29] Heo TH, Wahler J, Suh N. Potential therapeutic implications of IL-6/IL-6R/gp130-targeting agents in breast cancer. Oncotarget. 2016; 7:15460–73. 10.18632/oncotarget.7102 26840088 PMC4941253

[30] Johnson DE, O’Keefe RA, Grandis JR. Targeting the IL-6/JAK/STAT3 signalling axis in cancer. Nat Rev Clin Oncol. 2018; 15:234–48. 10.1038/nrclinonc.2018.8 29405201 PMC5858971

[31] Fassina A, Cappellesso R, Guzzardo V, Dalla Via L, Piccolo S, Ventura L, Fassan M. Epithelial-mesenchymal transition in malignant mesothelioma. Mod Pathol. 2012; 25:86–99. 10.1038/modpathol.2011.144 21983934

[32] Yu M, Bardia A, Wittner BS, Stott SL, Smas ME, Ting DT, Isakoff SJ, Ciciliano JC, Wells MN, Shah AM, Concannon KF, Donaldson MC, Sequist LV, et al. Circulating breast tumor cells exhibit dynamic changes in epithelial and mesenchymal composition. Science. 2013; 339:580–84. 10.1126/science.1228522 23372014 PMC3760262

[33] Aiello NM, Kang Y. Context-dependent EMT programs in cancer metastasis. J Exp Med. 2019; 216:1016–26. 10.1084/jem.20181827 30975895 PMC6504222

[34] Suarez-Carmona M, Lesage J, Cataldo D, Gilles C. EMT and inflammation: inseparable actors of cancer progression. Mol Oncol. 2017; 11:805–23. 10.1002/1878-0261.12095 28599100 PMC5496491

[35] Jiang YX, Yang SW, Li PA, Luo X, Li ZY, Hao YX, Yu PW. The promotion of the transformation of quiescent gastric cancer stem cells by IL-17 and the underlying mechanisms. Oncogene. 2017; 36:1256–64. 10.1038/onc.2016.291 27524415 PMC5340802

[36] Fernando RI, Hamilton DH, Dominguez C, David JM, McCampbell KK, Palena C. IL-8 signaling is involved in resistance of lung carcinoma cells to erlotinib. Oncotarget. 2016; 7:42031–44. 10.18632/oncotarget.9662 27248176 PMC5173114

[37] Gautier L, Cope L, Bolstad BM, Irizarry RA. affy—analysis of Affymetrix GeneChip data at the probe level. Bioinformatics. 2004; 20:307–15. 10.1093/bioinformatics/btg405 14960456

[38] Huang DW, Sherman BT, Tan Q, Collins JR, Alvord WG, Roayaei J, Stephens R, Baseler MW, Lane HC, Lempicki RA. The DAVID Gene Functional Classification Tool: a novel biological module-centric algorithm to functionally analyze large gene lists. Genome Biol. 2007; 8:R183. 10.1186/gb-2007-8-9-r183 17784955 PMC2375021

[39] Kanehisa M. The KEGG database. Novartis Found Symp. 2002; 247:91–101; discussion 101–3, 119–28, 244–52. 10.1002/0470857897.ch8 12539951

[40] Ashburner M, Ball CA, Blake JA, Botstein D, Butler H, Cherry JM, Davis AP, Dolinski K, Dwight SS, Eppig JT, Harris MA, Hill DP, Issel-Tarver L, et al. Gene ontology: tool for the unification of biology. Nat Genet. 2000; 25:25–9. 10.1038/75556 10802651 PMC3037419

[41] Szklarczyk D, Franceschini A, Wyder S, Forslund K, Heller D, Huerta-Cepas J, Simonovic M, Roth A, Santos A, Tsafou KP, Kuhn M, Bork P, Jensen LJ, von Mering C. STRING v10: protein-protein interaction networks, integrated over the tree of life. Nucleic Acids Res. 2015; 43:D447–52. 10.1093/nar/gku1003 25352553 PMC4383874

[42] Smoot ME, Ono K, Ruscheinski J, Wang PL, Ideker T. Cytoscape 2.8: new features for data integration and network visualization. Bioinformatics. 2011; 27:431–32. 10.1093/bioinformatics/btq675 21149340 PMC3031041

[43] Bandettini WP, Kellman P, Mancini C, Booker OJ, Vasu S, Leung SW, Wilson JR, Shanbhag SM, Chen MY, Arai AE. MultiContrast Delayed Enhancement (MCODE) improves detection of subendocardial myocardial infarction by late gadolinium enhancement cardiovascular magnetic resonance: a clinical validation study. J Cardiovasc Magn Reson. 2012; 14:83. 10.1186/1532-429X-14-83 23199362 PMC3552709

[44] Cerami E, Gao J, Dogrusoz U, Gross BE, Sumer SO, Aksoy BA, Jacobsen A, Byrne CJ, Heuer ML, Larsson E, Antipin Y, Reva B, Goldberg AP, et al. The cBio cancer genomics portal: an open platform for exploring multidimensional cancer genomics data. Cancer Discov. 2012; 2:401–04. 10.1158/2159-8290.CD-12-0095 22588877 PMC3956037

[45] Bindea G, Mlecnik B, Hackl H, Charoentong P, Tosolini M, Kirilovsky A, Fridman WH, Pagès F, Trajanoski Z, Galon J. ClueGO: a Cytoscape plug-in to decipher functionally grouped gene ontology and pathway annotation networks. Bioinformatics. 2009; 25:1091–93. 10.1093/bioinformatics/btp101 19237447 PMC2666812

[46] Bindea G, Galon J, Mlecnik B. CluePedia Cytoscape plugin: pathway insights using integrated experimental and in silico data. Bioinformatics. 2013; 29:661–63. 10.1093/bioinformatics/btt019 23325622 PMC3582273

[47] Subramanian A, Tamayo P, Mootha VK, Mukherjee S, Ebert BL, Gillette MA, Paulovich A, Pomeroy SL, Golub TR, Lander ES, Mesirov JP. Gene set enrichment analysis: a knowledge-based approach for interpreting genome-wide expression profiles. Proc Natl Acad Sci USA. 2005; 102:15545–50. 10.1073/pnas.0506580102 16199517 PMC1239896

[48] Xu WH, Xu Y, Wang J, Wan FN, Wang HK, Cao DL, Shi GH, Qu YY, Zhang HL, Ye DW. Prognostic value and immune infiltration of novel signatures in clear cell renal cell carcinoma microenvironment. Aging (Albany NY). 2019; 11:6999–7020. 10.18632/aging.102233 31493764 PMC6756904

[49] Higgins JP, Shinghal R, Gill H, Reese JH, Terris M, Cohen RJ, Fero M, Pollack JR, van de Rijn M, Brooks JD. Gene expression patterns in renal cell carcinoma assessed by complementary DNA microarray. Am J Pathol. 2003; 162:925–32. 10.1016/S0002-9440(10)63887-4 12598325 PMC1868114

[50] Yusenko MV, Kuiper RP, Boethe T, Ljungberg B, van Kessel AG, Kovacs G. High-resolution DNA copy number and gene expression analyses distinguish chromophobe renal cell carcinomas and renal oncocytomas. BMC Cancer. 2009; 9:152. 10.1186/1471-2407-9-152 19445733 PMC2686725

[51] Jones J, Otu H, Spentzos D, Kolia S, Inan M, Beecken WD, Fellbaum C, Gu X, Joseph M, Pantuck AJ, Jonas D, Libermann TA. Gene signatures of progression and metastasis in renal cell cancer. Clin Cancer Res. 2005; 11:5730–39. 10.1158/1078-0432.CCR-04-2225 16115910

[52] Gumz ML, Zou H, Kreinest PA, Childs AC, Belmonte LS, LeGrand SN, Wu KJ, Luxon BA, Sinha M, Parker AS, Sun LZ, Ahlquist DA, Wood CG, Copland JA. Secreted frizzled-related protein 1 loss contributes to tumor phenotype of clear cell renal cell carcinoma. Clin Cancer Res. 2007; 13:4740–49. 10.1158/1078-0432.CCR-07-0143 17699851

