# Supplementary material for: Identification of potential novel differentially-expressed genes and their role in invasion and migration in renal cell carcinoma
Source: Aging (Albany NY). 2020 May 18;12(10):9205–23. doi: 10.18632/aging.103192 (PMC7288970; doi:10.18632/aging.103192)
Supplement: Supplementary Table 1 [file aging-12-103192-s001..docx]

Supplementary Table 1. Correlations between EMT genes and TIMP1 expression.

| GeneName | P.value.Spearman | Cor.value.Spearman | P.value.Pearson | Cor.value.pearson |
| --- | --- | --- | --- | --- |
| ABI3BP | 3.32E-28 | 0.8349137 | 2.01E-34 | 0.878065201 |
| ACTA2 | 1.24E-20 | 0.757897208 | 3.11E-19 | 0.739482208 |
| ADAM12 | 3.72E-38 | 0.89801634 | 5.40E-10 | 0.561963585 |
| ANPEP | 1.49E-16 | 0.699335579 | 2.37E-16 | 0.696047984 |
| APLP1 | 2.96E-25 | 0.808779637 | 2.64E-07 | 0.479298022 |
| BASP1 | 1.98E-45 | 0.927649882 | 8.62E-29 | 0.839613574 |
| BDNF | 2.14E-27 | 0.828154799 | 2.54E-07 | 0.479902587 |
| BGN | 5.93E-38 | 0.897029444 | 1.57E-27 | 0.829311525 |
| BMP1 | 1.72E-38 | 0.899632715 | 7.50E-52 | 0.946358857 |
| CADM1 | 2.09E-18 | 0.727827523 | 7.96E-16 | 0.687163759 |
| CALD1 | 3.32E-31 | 0.857505608 | 2.40E-19 | 0.741020173 |
| CALU | 7.28E-58 | 0.959371799 | 1.00E-10 | 0.581049954 |
| CAP2 | 5.79E-26 | 0.815453185 | 1.02E-33 | 0.873858873 |
| CAPG | 1.05E-35 | 0.885357403 | 1.55E-11 | 0.600840881 |
| CD44 | 5.23E-55 | 0.95365058 | 8.06E-19 | 0.733737048 |
| CD59 | 8.02E-26 | 0.814140881 | 1.26E-19 | 0.744799231 |
| CDH11 | 1.08E-49 | 0.940703577 | 2.51E-06 | 0.442821691 |
| CDH2 | 2.23E-32 | 0.865408416 | 1.65E-48 | 0.937340384 |
| CDH6 | 6.87E-33 | 0.868697538 | 5.19E-12 | 0.611858071 |
| COL11A1 | 4.89E-36 | 0.887160488 | 9.44E-52 | 0.946109436 |
| COL12A1 | 7.99E-32 | 0.861730588 | 4.10E-07 | 0.472508288 |
| COL16A1 | 4.49E-27 | 0.825396826 | 4.17E-05 | 0.390454139 |
| COL1A1 | 7.98E-59 | 0.961129649 | 1.66E-78 | 0.984194723 |
| COL1A2 | 1.98E-59 | 0.962196563 | 3.01E-33 | 0.870949414 |
| COL3A1 | 6.16E-54 | 0.951297905 | 3.57E-35 | 0.882391328 |
| COL4A1 | 2.93E-50 | 0.942242446 | 1.24E-89 | 0.99046477 |
| COL4A2 | 1.14E-48 | 0.937812073 | 6.31E-73 | 0.979620351 |
| COL5A1 | 1.18E-51 | 0.945867441 | 3.72E-53 | 0.949504484 |
| COL5A2 | 2.00E-53 | 0.950129764 | 4.82E-63 | 0.967978551 |
| COL5A3 | 4.45E-38 | 0.897637585 | 5.79E-51 | 0.944102435 |
| COL6A2 | 1.18E-55 | 0.955016231 | 7.78E-40 | 0.905835895 |
| COL6A3 | 4.18E-56 | 0.955939111 | 1.81E-52 | 0.947874084 |
| COL7A1 | 7.21E-24 | 0.794925756 | 1.15E-06 | 0.455962783 |
| COL8A2 | 9.28E-36 | 0.88564547 | 8.84E-21 | 0.759726531 |
| COMP | 1.47E-28 | 0.837778365 | 3.05E-07 | 0.477070334 |
| COPA | 1.23E-34 | 0.87931568 | 5.42E-27 | 0.824683057 |
| CRLF1 | 1.29E-24 | 0.802538189 | 3.96E-06 | 0.434938231 |
| CTHRC1 | 1.00E-54 | 0.953042439 | 3.89E-55 | 0.953924286 |
| CXCL1 | 2.10E-32 | 0.865576814 | 5.39E-20 | 0.749690912 |
| CXCL12 | 9.63E-18 | 0.718027916 | 0.000267595 | 0.35024534 |
| CXCL6 | 2.98E-22 | 0.777311003 | 0.015068053 | 0.237797977 |
| DAB2 | 6.11E-18 | 0.720993937 | 9.76E-20 | 0.74628907 |
| DCN | 6.90E-19 | 0.734687781 | 0.013818216 | 0.240761992 |
| DKK1 | 5.17E-30 | 0.848954291 | 0.000394681 | 0.341119714 |
| DPYSL3 | 4.90E-40 | 0.906727694 | 1.21E-17 | 0.71651473 |
| DST | 8.83E-22 | 0.771843068 | 6.84E-24 | 0.795158342 |
| ECM1 | 1.43E-28 | 0.837874387 | 2.51E-10 | 0.5707939 |
| ECM2 | 1.50E-20 | 0.75683692 | 1.06E-13 | 0.647836297 |
| EDIL3 | 8.50E-32 | 0.861549213 | 6.47E-12 | 0.609672088 |
| EFEMP2 | 4.48E-39 | 0.902380019 | 2.76E-33 | 0.871186936 |
| ELN | 1.71E-19 | 0.743025715 | 1.79E-06 | 0.448636872 |
| EMP3 | 1.82E-60 | 0.963956972 | 1.75E-20 | 0.75599238 |
| ENO2 | 7.50E-45 | 0.925660087 | 2.76E-26 | 0.818400957 |
| FAP | 2.05E-46 | 0.930909304 | 5.31E-31 | 0.856082676 |
| FAS | 2.75E-49 | 0.939572648 | 1.97E-23 | 0.790317434 |
| FBLN1 | 1.13E-21 | 0.770562771 | 0.000819583 | 0.323166914 |
| FBLN2 | 1.02E-38 | 0.900710298 | 2.63E-14 | 0.659665959 |
| FBLN5 | 3.63E-15 | 0.675607408 | 0.001226548 | 0.312773492 |
| FBN1 | 1.65E-50 | 0.942906755 | 1.33E-38 | 0.900160255 |
| FBN2 | 5.64E-32 | 0.862744157 | 3.93E-12 | 0.61460216 |
| FERMT2 | 1.09E-18 | 0.73186241 | 1.68E-20 | 0.756213571 |
| FGF2 | 2.89E-34 | 0.877142164 | 9.12E-08 | 0.495153118 |
| FLNA | 2.77E-54 | 0.952071547 | 1.21E-13 | 0.646717937 |
| FMOD | 4.09E-32 | 0.863667038 | 4.23E-16 | 0.69184279 |
| FN1 | 1.14E-54 | 0.952919744 | 1.05E-79 | 0.985032835 |
| FOXC2 | 5.62E-25 | 0.806085679 | 1.00E-07 | 0.493747929 |
| FSTL1 | 4.90E-39 | 0.902198644 | 3.10E-74 | 0.980800653 |
| FSTL3 | 6.49E-37 | 0.89179623 | 2.26E-54 | 0.952266867 |
| FUCA1 | 1.33E-20 | 0.757498406 | 1.34E-05 | 0.412672182 |
| GADD45A | 1.60E-14 | 0.66376466 | 7.65E-22 | 0.772570374 |
| GADD45B | 1.73E-20 | 0.756042069 | 2.43E-22 | 0.778315078 |
| GAS1 | 6.39E-16 | 0.688794467 | 0.007172897 | 0.262189327 |
| GEM | 7.67E-38 | 0.896479984 | 1.10E-11 | 0.604322689 |
| GJA1 | 7.88E-48 | 0.935326329 | 3.86E-39 | 0.902678371 |
| GLIPR1 | 1.39E-57 | 0.958841118 | 7.85E-46 | 0.928996534 |
| GPC1 | 1.34E-31 | 0.860210236 | 1.36E-24 | 0.802298343 |
| GPX7 | 5.54E-35 | 0.881308464 | 2.14E-24 | 0.800316976 |
| GREM1 | 2.74E-27 | 0.827237253 | 4.24E-08 | 0.506103297 |
| HTRA1 | 5.63E-26 | 0.815565211 | 3.27E-10 | 0.567787763 |
| ID2 | 7.41E-32 | 0.861949306 | 7.26E-32 | 0.862008122 |
| IGFBP2 | 2.36E-15 | 0.678920176 | 0.002685167 | 0.291441274 |
| IGFBP3 | 8.41E-42 | 0.914196094 | 1.54E-54 | 0.952633385 |
| IGFBP4 | 1.02E-18 | 0.732294512 | 1.98E-14 | 0.662019173 |
| IL15 | 8.76E-46 | 0.928839491 | 5.94E-60 | 0.963094722 |
| IL32 | 3.07E-33 | 0.870895381 | 1.32E-42 | 0.917385174 |
| IL6 | 8.53E-26 | 0.813895491 | 0.353894131 | 0.091823509 |
| INHBA | 4.07E-36 | 0.887592588 | 8.90E-44 | 0.92181643 |
| ITGA2 | 5.08E-21 | 0.762710282 | 6.58E-08 | 0.499870334 |
| ITGA5 | 4.34E-51 | 0.944427107 | 3.88E-70 | 0.976854945 |
| ITGAV | 1.28E-34 | 0.879217312 | 7.39E-15 | 0.669990337 |
| ITGB1 | 5.89E-49 | 0.938633763 | 2.82E-45 | 0.927124241 |
| ITGB3 | 5.05E-21 | 0.762736955 | 1.94E-09 | 0.546639742 |
| ITGB5 | 3.90E-23 | 0.787137283 | 1.15E-13 | 0.647156162 |
| JUN | 3.96E-31 | 0.856969102 | 7.54E-74 | 0.980459753 |
| LAMA1 | 5.99E-21 | 0.761830078 | 6.28E-13 | 0.631972594 |
| LAMA2 | 7.43E-21 | 0.760667142 | 0.001298306 | 0.311277536 |
| LAMA3 | 2.45E-21 | 0.766556508 | 7.57E-30 | 0.847725536 |
| LAMC1 | 5.54E-47 | 0.932717724 | 1.50E-14 | 0.664303503 |
| LAMC2 | 1.91E-21 | 0.767874147 | 3.75E-06 | 0.435864486 |
| LGALS1 | 1.86E-58 | 0.960467951 | 6.87E-49 | 0.938441884 |
| LOX | 3.93E-49 | 0.939135213 | 2.62E-63 | 0.968365079 |
| LOXL1 | 6.43E-25 | 0.80551488 | 3.61E-09 | 0.538874091 |
| LOXL2 | 5.51E-28 | 0.833105281 | 1.35E-58 | 0.960719183 |
| LRP1 | 4.08E-37 | 0.892830851 | 5.04E-38 | 0.897375298 |
| LRRC15 | 1.47E-25 | 0.81166564 | 1.08E-10 | 0.580236682 |
| LUM | 2.01E-26 | 0.819640823 | 0.002506365 | 0.293382439 |
| MAGEE1 | 1.41E-14 | 0.664788898 | 0.039672257 | 0.202075972 |
| MATN3 | 3.75E-21 | 0.764321323 | 0.120322904 | 0.153276267 |
| MCM7 | 2.09E-43 | 0.920442876 | 4.59E-42 | 0.915254483 |
| MFAP5 | 2.13E-28 | 0.836476729 | 3.19E-07 | 0.476404235 |
| MGP | 3.40E-21 | 0.764838776 | 4.07E-09 | 0.537343039 |
| MMP1 | 8.75E-29 | 0.839560111 | 4.31E-63 | 0.96804997 |
| MMP14 | 1.27E-40 | 0.909288288 | 1.21E-96 | 0.993060305 |
| MMP2 | 3.17E-27 | 0.826698461 | 2.26E-15 | 0.679257146 |
| MMP3 | 1.08E-22 | 0.782288158 | 7.17E-14 | 0.651222502 |
| MSX1 | 4.60E-25 | 0.806928541 | 0.426136465 | -0.078868162 |
| MXRA5 | 7.94E-22 | 0.772381859 | 2.15E-25 | 0.810114894 |
| MYL9 | 5.58E-27 | 0.824575302 | 1.38E-08 | 0.521446199 |
| MYLK | 2.00E-22 | 0.77927946 | 1.69E-11 | 0.599937291 |
| NID2 | 3.81E-40 | 0.907207806 | 1.37E-36 | 0.890110245 |
| NNMT | 9.58E-48 | 0.935070097 | 2.90E-44 | 0.923586591 |
| NOTCH2 | 2.67E-43 | 0.920045237 | 4.56E-33 | 0.869825486 |
| NT5E | 1.23E-20 | 0.75794651 | 8.07E-06 | 0.422127506 |
| NTM | 4.39E-20 | 0.750856866 | 0.000137009 | 0.365347756 |
| OXTR | 1.09E-33 | 0.873674693 | 8.42E-08 | 0.496312777 |
| PCOLCE | 2.21E-41 | 0.912478362 | 9.72E-38 | 0.895969639 |
| PCOLCE2 | 1.09E-20 | 0.758581324 | 7.69E-18 | 0.719496099 |
| PDGFRB | 1.18E-10 | 0.579239928 | 0.036758559 | -0.20508454 |
| PDLIM4 | 6.11E-24 | 0.795672596 | 8.98E-15 | 0.66843062 |
| PFN2 | 7.61E-17 | 0.704067344 | 2.24E-09 | 0.544844616 |
| PLAUR | 1.03E-49 | 0.940756922 | 7.47E-48 | 0.935395893 |
| PLOD1 | 9.88E-36 | 0.885496102 | 2.24E-82 | 0.986744264 |
| PLOD2 | 2.86E-53 | 0.949772347 | 6.12E-106 | 0.995444345 |
| PLOD3 | 5.31E-47 | 0.932776404 | 1.00E-57 | 0.959111851 |
| PMEPA1 | 2.01E-46 | 0.930935977 | 2.01E-52 | 0.947759608 |
| PMP22 | 4.15E-33 | 0.870079192 | 3.14E-12 | 0.616778278 |
| POSTN | 2.72E-38 | 0.898677827 | 1.83E-05 | 0.406779838 |
| PPIB | 2.40E-41 | 0.912334328 | 4.27E-17 | 0.708058637 |
| PRRX1 | 2.53E-37 | 0.893892717 | 7.61E-49 | 0.938315563 |
| PTHLH | 1.27E-38 | 0.90025686 | 2.56E-18 | 0.726554323 |
| PTX3 | 6.81E-30 | 0.848068752 | 0.000699489 | 0.327153031 |
| PVR | 3.45E-29 | 0.842718177 | 2.78E-41 | 0.912065342 |
| QSOX1 | 1.03E-42 | 0.917807598 | 4.53E-12 | 0.61319843 |
| RGS4 | 6.65E-34 | 0.874976328 | 1.62E-15 | 0.681809171 |
| RHOB | 4.52E-27 | 0.825364818 | 1.01E-30 | 0.854120306 |
| SAT1 | 2.37E-19 | 0.741107842 | 3.80E-46 | 0.930037602 |
| SCG2 | 2.76E-38 | 0.898640485 | 8.69E-11 | 0.58261076 |
| SDC1 | 1.85E-16 | 0.697825896 | 8.78E-08 | 0.495694026 |
| SDC4 | 4.79E-15 | 0.673430903 | 3.53E-14 | 0.657225895 |
| SERPINE1 | 5.15E-47 | 0.932819081 | 2.97E-67 | 0.973594796 |
| SERPINE2 | 9.49E-31 | 0.854299531 | 4.61E-35 | 0.881761879 |
| SERPINH1 | 8.13E-60 | 0.962863384 | 8.20E-83 | 0.987004417 |
| SFRP1 | 1.07E-14 | 0.667029418 | 0.016893952 | 0.233830189 |
| SFRP4 | 5.33E-34 | 0.875552462 | 1.68E-07 | 0.486184301 |
| SGCB | 1.76E-27 | 0.82889097 | 6.75E-16 | 0.688394322 |
| SGCD | 8.18E-20 | 0.747309376 | 0.000130032 | 0.366495964 |
| SGCG | 5.40E-34 | 0.875517455 | 0.002133776 | 0.297865148 |
| SLC6A8 | 7.50E-31 | 0.855025033 | 2.16E-88 | 0.989912133 |
| SLIT2 | 3.54E-16 | 0.693126139 | 1.00E-05 | 0.418144425 |
| SLIT3 | 1.37E-22 | 0.781141225 | 2.06E-15 | 0.679968242 |
| SNAI2 | 4.64E-28 | 0.833724091 | 1.01E-12 | 0.627610278 |
| SNTB1 | 5.21E-26 | 0.815879951 | 4.01E-08 | 0.506863417 |
| SPARC | 3.92E-48 | 0.936233206 | 6.32E-15 | 0.671233108 |
| SPOCK1 | 7.10E-24 | 0.794995106 | 5.33E-17 | 0.706530543 |
| SPP1 | 6.94E-24 | 0.795096463 | 4.49E-15 | 0.673934545 |
| TAGLN | 1.58E-27 | 0.829280393 | 2.99E-06 | 0.439844505 |
| TFPI2 | 1.65E-39 | 0.904369814 | 6.20E-11 | 0.586268142 |
| TGFB1 | 5.84E-34 | 0.875314741 | 2.13E-28 | 0.836482924 |
| TGFBI | 2.20E-54 | 0.952295599 | 3.18E-72 | 0.978956939 |
| TGFBR3 | 4.86E-14 | 0.654530518 | 4.52E-08 | 0.505192717 |
| TGM2 | 1.87E-44 | 0.924262429 | 2.06E-26 | 0.819544519 |
| THBS1 | 1.04E-26 | 0.822185414 | 1.02E-25 | 0.813172361 |
| THBS2 | 5.61E-50 | 0.941482424 | 3.16E-29 | 0.843019041 |
| THY1 | 1.01E-16 | 0.702068752 | 3.03E-31 | 0.857778827 |
| TIMP1 | 0 | 1 | 0 | 1 |
| TIMP3 | 1.99E-20 | 0.75527389 | 1.50E-11 | 0.601156698 |
| TNC | 2.91E-26 | 0.818195155 | 0.00040604 | 0.340442423 |
| TNFAIP3 | 4.65E-45 | 0.926380254 | 6.74E-66 | 0.971903864 |
| TNFRSF11B | 1.53E-20 | 0.756751566 | 8.14E-11 | 0.583330449 |
| TNFRSF12A | 9.69E-39 | 0.900811655 | 4.41E-22 | 0.77535345 |
| TPM1 | 4.18E-38 | 0.89777095 | 2.98E-54 | 0.952001943 |
| TPM2 | 1.81E-32 | 0.865992911 | 6.17E-19 | 0.735368467 |
| TPM4 | 8.16E-42 | 0.914249439 | 1.39E-16 | 0.699821754 |
| VCAM1 | 4.43E-40 | 0.906919739 | 7.16E-39 | 0.901429192 |
| VCAN | 4.18E-58 | 0.959819905 | 1.14E-30 | 0.853734699 |
| VEGFA | 1.20E-35 | 0.885031994 | 3.74E-60 | 0.963435358 |
| VEGFC | 6.26E-41 | 0.910589924 | 2.10E-38 | 0.899212503 |
| VIM | 7.33E-49 | 0.9383617 | 8.44E-25 | 0.804355928 |
| WIPF1 | 2.65E-45 | 0.927217781 | 7.63E-77 | 0.982951668 |
| WNT5A | 1.95E-29 | 0.844627954 | 5.93E-07 | 0.466677083 |
